# The effect of vitamin D deficiency in children with overactive bladder related urinary incontinence

**DOI:** 10.1590/S1677-5538.IBJU.2021.0645

**Published:** 2022-01-10

**Authors:** Burak Özçift, Uygar Micoogullari

**Affiliations:** 1 Health Sciences University Child Diseases and Surgery Training and Research Hospital - Pediatric Urology Izmir Turkey Health Sciences University, Izmir Dr. Behcet Uz Child Diseases and Surgery Training and Research Hospital - Pediatric Urology Izmir, Turkey; 2 Izmir Tepecik Training and Research Hospital - Urology Konak Izmir Turkey Izmir Tepecik Training and Research Hospital - Urology Konak, Izmir, Turkey

**Keywords:** Urinary Bladder, Overactive, Quality of Life, Urinary Incontinence

## Abstract

**Purpose:**

Overactive bladder (OAB) is a common syndrome associated with lower urinary tract symptoms (LUTS), especially urinary incontinence in children, which may affect the patient's quality of life (QoL). Vitamin D deficiency has been shown to be associated with OAB syndrome. This study evaluated the relationship between vitamin D status and OAB-related symptoms and QoL in children.

**Materials and Methods:**

The study included 52 pediatric patients with OAB-related urinary incontinence and 41 healthy children. LUTS were assessed using the Dysfunctional Voiding and Incontinence Symptoms Score (DVISS) questionnaire, and QoL was assessed using the Pediatric Incontinence Questionnaire (PINQ). Oral vitamin D supplementation was given to patients with OAB with vitamin D deficiency. Urinary symptoms and QoL were evaluated before and after vitamin D supplementation.

**Results:**

Vitamin D deficiency was more common in the OAB group (75%) than in the control group (36.6%). Logistic regression analysis revealed that vitamin D status (<20ng/mL) was a significant predictor of OAB. Both pre-treatment and post-treatment DVISS and PINQ scores showed a positive correlation. After vitamin D supplementation, 8 (23.5%) patients had a complete response and 19 (55.9%) patients had a partial response. Significant improvement in QoL was also achieved.

**Conclusions:**

Vitamin D deficiency is more common in children with urinary incontinence and OAB than in healthy children. Although vitamin D deficiency is not routinely evaluated for every patient, it should be evaluated in treatment-resistant OAB cases. Vitamin D supplementation may improve urinary symptoms and QoL in patients with OAB.

## INTRODUCTION

According to the International Children's Continence Society (ICCS), overactive bladder (OAB) is characterized by urinary urgency and may be associated with daytime incontinence, frequency, and holding maneuvers used to prevent involuntary urinary incontinence (UI) (1). It is the most common cause of voiding dysfunction in children and is defined as bladder storage phase dysfunction (2). OAB is not synonymous with detrusor overactivity, because although detrusor overactivity is a urodynamic diagnosis, OAB is a symptom-based diagnosis (3). OAB is commonly found in children (especially between ages 5-7 years and in boys) and adolescents, affecting around 5-12% of children aged between 5 and 10 years and 0.5% of adolescents up to age 18 years with daytime symptoms (2, 4).

Clinically, urgency is usually associated with UI and frequency (1, 2). UI is seen in around 14-30% of OAB cases (4, 5). UI impairs quality of life (QoL) and negatively affects children's social, behavioral, and emotional well-being. These issues tend to improve after successful treatment of UI (6). Treatment options for OAB tend to be staggered, with “lines of therapy” that correspond to different levels of invasiveness ranging from least to most invasive (7).

Lower urinary tract symptoms (LUTS) may be a result of multifactorial causes; OAB in particular is a well-known cause of LUTS. To prevent the occurrence of LUTS and reduce their severity, the relationship between many micronutrients and OAB has been widely assessed and the growing number of studies recently suggested that serum vitamin D deficiency was associated with OAB. This may be due to the effects of vitamin D on muscle function through the vitamin D receptors distributed throughout the bladder wall. Vitamin D deficiency or insufficiency may result in abnormalities in calcium homeostasis with ensuing abnormal detrusor contractility because the active metabolite acts through the vitamin D receptor. Weakened detrusor muscles may also become hypercontractile or irritable similar to that seen in hypocalcemic skeletal muscle function (8). It has been reported in previous studies that serum vitamin D levels are lower in patients with OAB and that vitamin D supplementation as an alternative treatment option reduces OAB-related symptoms and improved QoL in adults (9, 10).

Vitamin D deficiency in children can cause OAB through detrusor muscle activity and impair QoL, especially by causing UI. Vitamin D intake can also reduce symptoms and improve QoL, as in adults. As far as we know, there is no study in the literature evaluating the relationship between vitamin D and OAB in the pediatric age group. In this study, we aimed to analyze serum vitamin D levels in children with OAB-related UI, to determine their effect on symptoms and QoL, and to evaluate whether vitamin D supplementation alleviated these symptoms and improved QoL.

## MATERIALS AND METHODS

This study was prospectively planned and conducted at the Pediatric Urology Clinic of Behcet Uz Training and Research Hospital between May 2017 and July 2021. Only children aged over 5 years who presented to the pediatric urology outpatient clinic were included in the study. Participants were divided into two groups as patients with OAB-related UI (group 1) and a healthy control group (group 2). The study followed the principles of the latest edition of the Declaration of Helsinki guidelines, written and verbal informed consent was obtained from all participants, and the institution's internal review board approved the study protocol (605-2021/14-06).

Based on the ICCS criteria, OAB was clinically defined as (I) urinary urgency with or without urge incontinence, (II) need for holding maneuver, (III) urination more than seven times a day. Findings supporting the diagnosis of OAB were normal or low bladder capacity, a tower-shaped voiding curve in uroflowmetry, and post-void residual urine volume <20mL (1).

Patients with clinically diagnosed OAB-related UI who did not benefit from the use of anticholinergics after standard urotherapy and were referred for further evaluation were included in the OAB-related UI study group. The criteria for inclusion in the control group were a Dysfunctional Voiding and Incontinence Symptoms Score (DVISS) of 0, and no indication of constipation (Rome III). Patients with kidney and liver disease, genetic and muscle diseases, chronic diseases affecting vitamin D metabolism, neurologic disease, history of urologic surgery, urologic malignancies, psychiatric disorders, recurrent urinary tract infections, and those taking any medication that might affect LUTS or vitamin D levels were excluded from the study.

**Figure 1:**
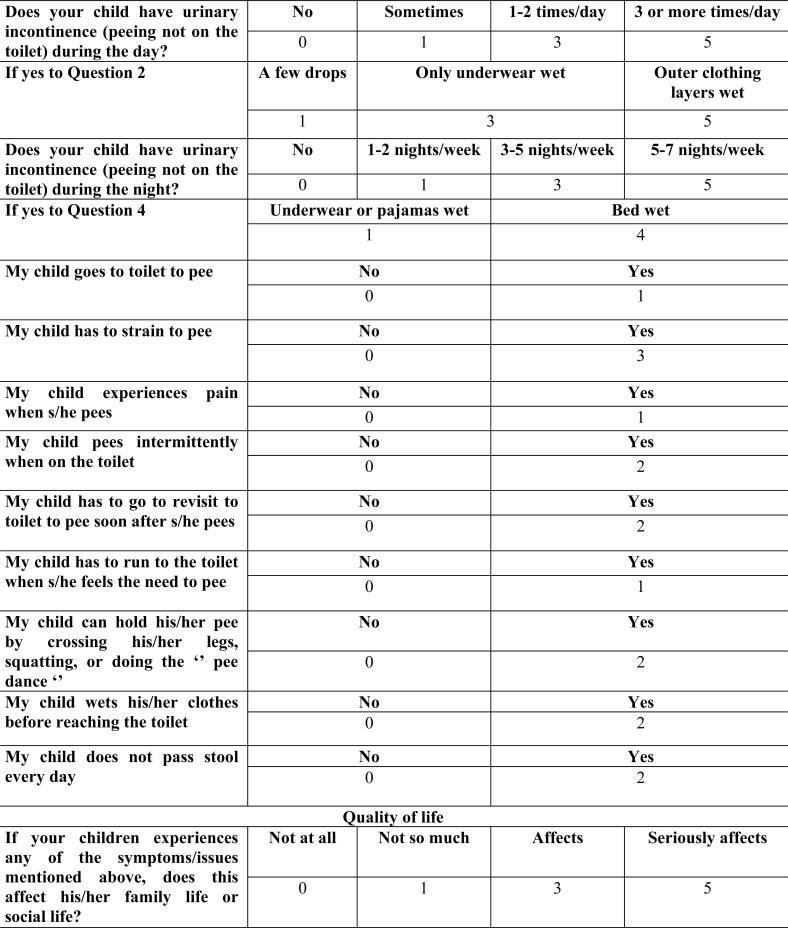
The Dysfunctional Voiding and Incontinence Symptoms Score (DVISS) form for children.

**Figure 2:**
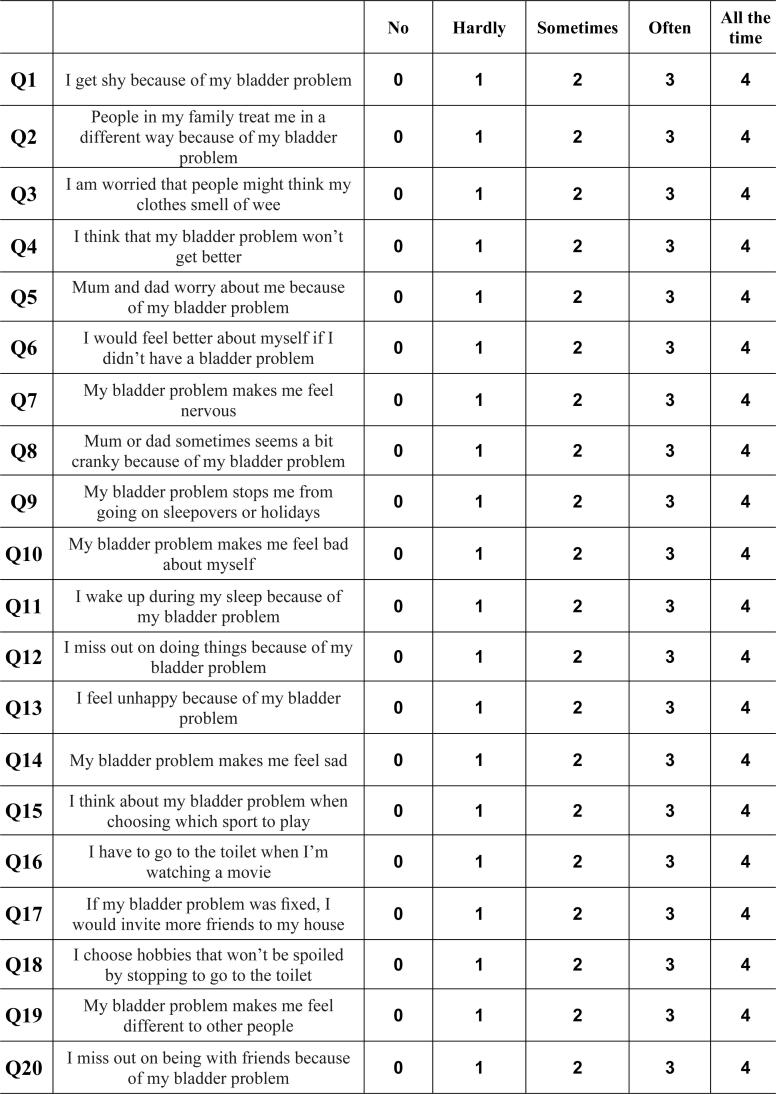
The Pediatric Incontinence Questionnaire (PINQ) form for children.

The initial evaluation of the participants began by taking their medical histories. In addition to the demographic data of the patients, symptoms such as daytime urinary frequency, sense of urgency, nocturia, enuresis, and urinary incontinence were recorded. Each patient completed at least a 48-hours bladder diary according to the recommendations of the ICCS (1). Uroflowmetry was performed in all patients in the patient group, and post-void residual urine was evaluated with ultrasound. LUTS were evaluated using the DVISS (Figure-1). QoL was evaluated using question 14 of the DVISS (11) and the Pediatric Incontinence Questionnaire (PINQ) (Figure-2) (12, 13). Participant's blood levels of 25 (OH)D were examined. Vitamin D deficiency was considered as <20ng/mL (14).

### Patient follow-up

Patients with OAB and no vitamin D deficiency were referred for biofeedback and transcutaneous electrical nerve stimulation (TENS) therapy and were not included in the further course of the study.

Only oral vitamin D3 2000 IU/day was prescribed for 8 weeks (loading dose) to participants with OAB and vitamin D deficiency (14). DVISS, QoL, and PINQ scores and symptoms were recorded two months after the treatment in those who regularly used their prescribed vitamin D3. Response to treatment was evaluated as recommended by the ICCS (no response if there was <50% reduction, partial response if there was 50% to 99% reduction, and complete response if there was 100% reduction) (1).

The groups were evaluated before treatment concerning the sociodemographic data and vitamin D status. After vitamin D loading treatment, clinical response was evaluated within the patient group based on a reduction in clinical symptoms, DVISS scores, QoL scores, PINQ scores, and data from the bladder diary.

### Statistical Analysis

Statistical analyses were performed using the SPSS version 17.0 software package (SPSS Inc., Chicago IL, USA). The distribution of data was analyzed using the Kolmogorov-Smirnov and Shapiro-Wilk test. Continuous variables with normal distribution are presented as mean±standard deviation (SD) and those with non-normal distribution as median. Categorical data are reported as numbers (frequency). Student's t-test and the Chi-square test (or other appropriate non-parametric tests) were used to compare data. Pearson's correlation analysis was used to evaluate the relationship between vitamin D levels and selected parameters among patients with OAB for normally distributed data (Spearman's correlation analysis for non-normally distributed data). The McNemar test was used to compare the frequency of urinary symptoms before and after vitamin D treatment. The paired-sample t-test was used to compare PINQ and DVISS scores before and after vitamin D treatment. Binary logistic regression analysis was used to determine predictive factors for the development of OAB-related UI. P-values of <0.05 were considered statistically significant.

## RESULTS

This study included 52 patients in group 1 and 41 healthy children in group 2. There was no significant difference between the mean ages (P=0.08). Boys constituted 48.1% (n=25) of group 1 and 56.1% (n=23) of group 2 (P=0.44). Group 1 had lower vitamin D levels compared with group 2 (P=0.046). Vitamin D deficiency (<20ng/mL) was observed in 75% (n=39) of group 1 and 36.6% (n=15) of group 2 (P <0.001). Other characteristics of the participants are summarized in Table-1.

**Table 1:** Demographic and clinical characteristics of the study population.

		OAB (n=52)	Controls (n=41)	p value*
Mean age ± SD (years) (min-max)		7.71 ± 2.66 (5-16)	8.68 ± 2.48 (5-16)	0.08
Sex (n) (boy/girl)		25/27	23/18	0.44
Serum vitamin D ± SD (ng/mL)		17.10 ± 7.54	20.66 ± 9.46	0.046
**Serum vitamin D**				< 0.001
	Normal (≥ 20 ng/mL) (n) (%)	13 (25.0)	26 (63.4)	
	Deficiency (< 20 ng/mL) (n) (%)	39 (75.0)	15 (36.6)	
**Symptoms (n) (%)**				
	Frequency**	52 (100)	0 (0.00)	
	Urinary incontinence	52 (100)	0 (0.00)	
	Urgency	48 (92.3)	0 (0.00)	
	Enuresis	42 (80.0)	0 (0.00)	
	Holding maneuver	37 (71.2)	0 (0.00)	
	Constipation	17 (32.7)	0 (0.00)	

* P < 0.05 was considered statistically significant;

** > 7 times/day; OAB: overactive bladder

**Figure 3:**
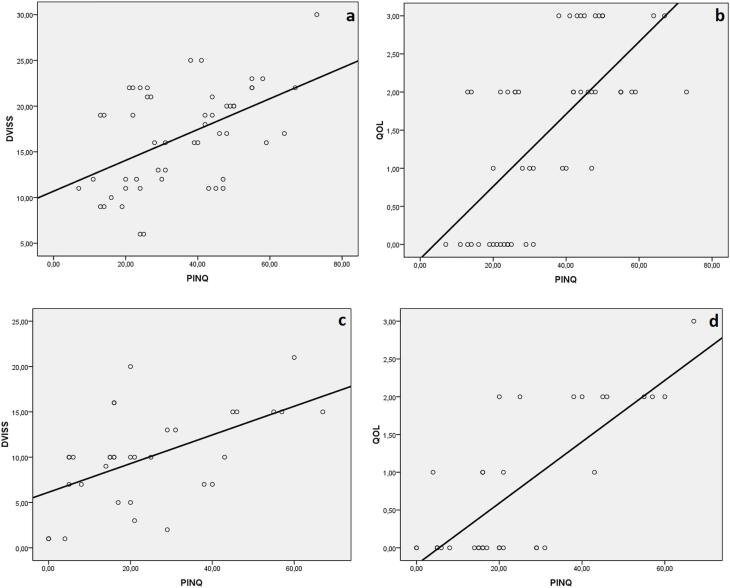
Scatter plot with trend line of patients with overactive bladder-related urinary incontinence before and after vitamin D treatment.

Correlation analysis was performed to evaluate the relationships between age, vitamin D levels, DVISS scores, QoL scores, and PINQ scores. None of the scoring systems had a significiant relationship with vitamin D levels and age (P >0.05). Pre-treatment DVISS scores correlated positively with QoL (r=0.584, P <0.001) and PINQ scores (r=0.455, P=0.001). PINQ scores were positively correlated with QoL scores (r=0.680, P <0.001). Post-treatment DVISS scores were also positively and significantly correlated with QOL (r=0.476, P=0.004) and PINQ scores (r=0.500, P=0.003). Moreover, PINQ scores had a significant positive correlation with QoL scores (r=0.681, P <0.001) (Figure-3). In multivariate analysis, vitamin D deficiency (<20ng/mL) (OR: 5.20, 95% CI: [2.13-12.70]; P <0.001) was found as an important factor for OAB-related UI, whereas sex (OR: 0.74, 95% CI: [0.32-1.65], P=0.19) was not found as a decisive factor for OAB-related UI.

The patients who had vitamin D deficiency and used regular vitamin D3 supplementation (n=34) had significantly lower DVISS, QoL, and PINQ scores compared with baseline, two months after the first dose of the supplement. Symptoms of urinary incontinence, urinary frequency, sense of urgency, and holding maneuver improved significantly after taking vitamin D supplementation (P <0.05) (Table-2). When we evaluated response to treatment as recommended by the ICCS, 8 (23.5%) patients had a complete response to vitamin D supplementation and 19 (55.9%) had a partial response. Seven (20.6%) patients did not respond to vitamin D supplementation. Patients were also evaluated according to preschool age-school age and sex in terms of response to treatment. While 77.8% (n=14) of preschool-aged children (5-7 years) and 81.3% (n=13) of school-aged children (≥7 years) responded to treatment (P=0.803), 80% (n=12) of boys and 78.9% (n=15) of girls responded to treatment (P=0.94).

**Table 2:** Effect of vitamin D supplementation on urinary symptoms and quality of life in patients who completed the follow-up questionnaire.

	Pre-treatment (n=34)	Post-treatment (n=34)	p value*
DVISS score (median [IQR])	17 (11.75-20.00)	10 (7.00-15.00)	**< 0.001**
QQL score (median [IQR])	2 (0-2)	0 (0-2)	**< 0.001**
PINQ score	33.62 ± 15.93	24.24 ± 18.16	**< 0.001**
Frequency (times/day) (median [IQR])	9 (8-10)	8 (6-10)	**< 0.001**
Urinary incontinence (n) (%)	34 (100)	18 (52.9)	**< 0.001**
Urgency (n) (%)	32 (94.1)	18 (52.9)	**< 0.001**
Enuresis (n) (%)	27 (79.4)	23 (67.6)	0.13
Urinary frequency (> 7 times/day) (n) (%)	34 (100)	17 (50.0)	**< 0.001**
Holding maneuver (n) (%)	24 (70.6)	11 (32.4)	**< 0.001**
Constipation (n) (%)	6 (17.6)	3 (8.8)	0.26

DVISS = Dysfunctional Voiding and Incontinence Symptoms Score Questionnaire; QOL = Quality of Life; PINQ = Pediatric Urinary Incontinence Quality of Life Score; IQR = Interquartile range

* P < 0.05 is considered statistically significant.

## DISCUSSION

Vitamin D is a pro-hormone that acts through its fat-soluble active metabolite 1.25 dihydroxy vitamin D (1.25 (OH) 2D) by binding to receptors in distant organs. In addition to calcium metabolism, it is effective in magnesium and phosphorus metabolism and many metabolic pathways in the body. Its active metabolite exerts its effect on vitamin D receptors (15, 16). Some studies have shown the presence of vitamin D receptors in the detrusor muscle and its significant effects, which can be explained by the importance of intracellular calcium concentration of vitamin D in the regulation of smooth muscle contractions (17, 18). It has also been suggested that the RhoA/Rho-related kinase pathway is responsible for the development of detrusor overactivity (19, 20). Vitamin D and its analogs have been shown to cause calcium insensitivity and relax smooth muscles (21). We planned this study considering the hypothesis that vitamin D might play a role in the pathophysiology of OAB and related symptoms.

Dallosso et al. reported that OAB symptoms were delayed by higher oral intake of protein, potassium, and vitamin D among various nutrients, and found that OAB symptoms were more common in people who were deficient in vitamin D intake (22). Digesu et al. presented the results of a study considering the role of a synthetic vitamin D analog in terms of treating UI. Cystometry testing was used to define bladder capacity before and after treatment. The bladder capacity in those receiving synthetic vitamin D analog treatment was superior to placebo. The authors emphasized that vitamin D analogs might be an alternative or supportive to anticholinergic agents (21).

Abdul-Razzak et al. reported lower levels of vitamin D in patients with OAB compared with a healthy control group. Vitamin D deficiency was more prevalent in patients with OAB (80%) than in controls (34.9%). They also showed that vitamin D supplementation improved UI and urinary symptoms in patients with OAB, as well as their QoL (23). Yoo et al. reported lower vitamin D levels in an adult patient group with LUTS and OAB and that their symptoms improved significantly with vitamin D supplementation. They associated the role of vitamin D in OAB symptoms with its effectiveness in detrusor muscle function, in particular (10). Kilic et al. reported that patients with OAB-related UI had lower vitamin levels than controls (19.16±9.08ng/mL vs. 21.25±13.64ng/mL; p=0.015) in the geriatric population. In another study, the authors reported two cases of UI whose symptoms resolved after vitamin D replacement (24). Parker et al. suggested that adequate vitamin D serum levels could cause an increase in pelvic floor muscle efficiency and a decrease in detrusor contraction strength resulting in a more effective response by reducing UI episodes compared with behavioral therapy. In conclusion, lower vitamin D levels were associated with worse QoL measures and LUTS (8). Badalian et al. and Vaughan et al. showed that higher vitamin D levels were preventive for UI in their studies (25, 26). In our study, the mean serum levels of vitamin D were only 2.5ng/mL higher in controls. Although this difference seems statistically significant, we thought that the fact that the higher number of patients with vitamin D deficiency in the OAB group created the clinical difference (75% vs. 36.6%, respectively). Moreover, we found that vitamin D deficiency increased the risk of OAB-related UI in children 3.5 times.

Children with UI have more social problems. Many studies have shown that children with UI have difficulties with self-confidence, social adaptation, behavioral problems with school and friends, and a decrease in QoL (13, 27). Bower et al. created the PINQ, as a cross-cultural tool specific to children with UI and lower urinary tract dysfunction. This questionnaire has proven to be a reliable and valid tool for children with UI and is recommended for assessing their QoL (13). Thibodeau et al. evaluated 40 children (10 males, 30 females) aged 5-11 years who had non-neurogenic UI and stated that PINQ and DVISS provided for making clinically appropriate assessments of LUTS and helped in their understanding of the impact of LUTS on QoL (28). Equit et al. also used this questionnaire to measure changes in QoL during treatment (29). In our study, the symptoms and QoL of children with OAB-related UI were evaluated using PINQ and DVISS, and it was shown that vitamin D supplementation significantly improved symptoms and QoL.

The limitations of the study were the small sample size, which could affect conclusions about the subgroup analyses, the patient group consisting of only those who used medication for OAB without clinical benefit, short-term follow-up and not measuring vitamin D levels after follow-up. In addition, we did not randomize patients when receiving placebo and vitamin D supplementation during the follow-up, but rather adopted a similar design to a previously published study (30). Despite these limitations, to the best of our knowledge, our study is the first to show that vitamin D supplementation can improve OAB and its associated symptoms in children, as well as QoL.

## CONCLUSION

In conclusion, this study showed that vitamin D deficiency was more common in children with OAB-related UI than in healthy children, and demonstrated that vitamin D supplementation could reduce urinary symptoms associated with OAB and improve QoL in treatment-resistant cases. These findings reflect the importance of evaluating vitamin D levels in children with OAB-related UI. Although vitamin D deficiency is not routinely evaluated in every patient, we suggest considering it in treatment-resistant cases. We think that vitamin D supplementation improves QoL with the improvement of symptoms and that vitamin D supplementation will increase the treatment response in treatment-resistant cases by supporting other treatment modalities.
